# Nutraceutical Characteristics of Ancient *Malus* x *domestica* Borkh. Fruits Recovered across Siena in Tuscany

**DOI:** 10.3390/medicines6010027

**Published:** 2019-02-18

**Authors:** Roberto Berni, Claudio Cantini, Massimo Guarnieri, Massimo Nepi, Jean-Francois Hausman, Gea Guerriero, Marco Romi, Giampiero Cai

**Affiliations:** 1 Department of Life Sciences, University of Siena, I-53100 Siena, Italy; berni10@student.unisi.it (R.B.); massimo.guarnieri@unisi.it (M.G.); massimo.nepi@unisi.it (M.N.); marco.romi@unisi.it (M.R.); 2 Trees and Timber Institute-National Research Council of Italy (CNR-IVALSA), I-58022 Follonica, Italy; cantini@ivalsa.cnr.it; 3 Environmental Research and Innovation Department, Luxembourg Institute of Science and Technology, L-4362 Esch/Alzette, Luxembourg; jean-francois.hausman@list.lu (J.-F.H.); gea.guerriero@list.lu (G.G.)

**Keywords:** *Malus* x *domestica*, Tuscany, ancient varieties, nutraceutics, antioxidants, polyphenols, sugars, pectin

## Abstract

**Background:** A diet rich in fruits and vegetables contributes to lowering the risk of chronic diseases. The fruits of Malus x domestica are a rich dietary source of bioactive compounds, namely vitamins and antioxidants, with recognized action on human health protection. Tuscany is known for its rich plant biodiversity, especially represented by ancient varieties of fruit trees. Particularly noteworthy are the many ancient Tuscan varieties of apple trees. **Methods:** Sugar quantification via HPLC and spectrophotometric assays to quantify the antioxidant power and total polyphenol content revealed interesting differences in 17 old varieties of Malus x domestica Borkh. recovered in Siena (Tuscany). **Results:** The quantification of antioxidants, polyphenols, and the main free sugars revealed that their content in the old fruits was often superior to the widespread commercial counterparts (‘Red Delicious’ and ‘Golden Delicious’). Such differences were, in certain cases, dramatic, with 8-fold higher values. Differences were also present for sugars and fibers (pectin). Most ancient fruits displayed low values of glucose and high contents of xylitol and pectin. **Conclusions:** The results reported here suggest the possible use of ancient apple varieties from Siena for nutraceutical purposes and draw attention to the valorization of local old varieties.

## 1. Introduction

The consumption of fruits and vegetables has beneficial effects on human health, as they contribute to lowering the risk of chronic diseases and improve the immune system [1].

The fruits of *Malus* x *domestica* are consumed worldwide [2,3] and are a rich source of phytochemicals (a.k.a. bioactive molecules that are plant metabolites with a biological effect) that contrast oxidative damage and positively impact human health [4]. In particular, polymeric apple proanthocyanidins contribute substantially to limiting lipid peroxidation and, therefore, oxidative stress [5]. Apple oligomeric procyanidins also display anti-cancer properties: they have anti-mutagenic effects, they modulate signal transduction pathways and they may even display epigenetic action [3,6]. 

The skins of apples contain pentacyclic triterpenes, which have anti-inflammatory effects [2]. Particularly noteworthy is the case of the triterpene-caffeates betulinic acid-3-*cis*-caffeic, betulinic acid-3-*trans*-caffeic and oleanolic acid-3-*trans*-caffeic reported in the suberized skin tissues of the russeted fruits of ‘Merton Russet’ [7]. The consumption of apple fruits with skins, therefore, results in a higher intake of bioactives, as well as of dietary fibers [8].

The concentration of bioactives varies in function of the apple fruit maturation stage and variety (reviewed by [3]). We have recently reported on the rich repertoire of ancient varieties of both herbaceous and woody plants of Tuscany. In particular, we have shown their nutraceutical values by measuring the content of key bioactive molecules (flavonoids, anthocyanins, carotenoids) [9,10]. Ancient varieties of plants were cultivated in the past but have fallen out of agricultural interest with the progressive development of cultivars meeting specific market needs, e.g., yield/key fruit characteristics such as shape, size and color. Interestingly, such ancient varieties display a high content of bioactive molecules (even superior than commercial counterparts) [9], as well as high adaptability to exogenous stresses [11]. These features make them interesting for agronomical and nutraceutical studies and as a source of interesting genetic characters to be used for breeding purposes. 

Many of these ancient varieties have been recovered across Tuscany and, subsequently, included in the regional germplasm bank through law 64/04 (recently reviewed by Berni and co-authors [11]). The province of Siena in Tuscany has recovered many plants thanks to the actions supported by this law, with the aim of protecting and propagating the old local germplasm. 

We here investigate the antioxidant power, as well as the polyphenol, free sugar and pectin content of 17 ancient apple fruits representing the biodiversity repertoire of Siena. We provide evidence of their value for nutraceutical applications and promote the valorization, on a local scale, of ancient plants for the diversification of the fruit market and their use in the daily diet as sources of functional molecules. 

Locally-grown ancient varieties will contribute to the restoration of regional habitats and exploit local resources, notably the soil (and associated microbial consortia). The products obtained by locally-grown ancient varieties are fully traceable, can be used as functional foods and are obtained with a “0 km” concept [9].

## 2. Materials and Methods 

### 2.1. Fruit Collection

The fruits of 17 ancient apples (a pool of 3–4 fruits per biological replicate) were harvested during the year 2014 from plants present in the experimental field “Il Campino”, a part of the Tuscan regional germplasm bank, localized in Siena (43°18′16″N 11°22′32″E). Fruits were collected at the maximum stage of maturation, i.e., between 30–40 days after flower anthesis. Our study included two commercial varieties ‘Red Delicious’ and ‘Golden Delicious’ for comparison. After harvest, fruits were immediately placed at −80 °C to block any metabolic processes. The ancient varieties studied here are: ‘Solaio’, ‘Campo Pianacce’, ‘Viale Casetta’, ‘Gialla Pianacce’, ‘Tre Colli’, ‘Ancaiano’, ‘Piatta Cantine’, ‘Filare Delle Pianacce’, ‘Tocchi’, ‘Rossa Casetta’, ‘Ficareto’, ‘Rugginosa Delle Pianacce’, ‘Vecchio Pollaio’, ‘Strada Pianacce’, ‘Podere Pianacce’, ‘Sotto Muro Casetta’, ‘Casolana’, ‘Red Delicious’, and ‘Golden Delicious’.

### 2.2. Extraction of Antioxidants and Phenolic Compounds

The extraction procedure was performed according to Berni et al. [12] following a previously described method [13]. Fruits were analyzed in three independent biological replicates, as well as three experimental replicates for each variety. Three grams of frozen fruits were added to 9.0 mL of 70% acetone and then homogenized using an Ultra-Turrax® T-25 basic (IKA®-Werke GmbH & Co., IKA, Staufen, Germany). The mixture was then sonicated for 20 min with an Elma Transsonic T 460/H for 20 min and homogenized again to ensure complete tissue lysis. The final mixture was centrifuged for 5 min at 12,000 rpm (centrifuge 5415D, Eppendorf®, Hamburg, Germany) and filtered through a 0.45 µm membrane to remove impurities.

### 2.3. Evaluation of the Antioxidant Power

The ferric reducing antioxidant power (FRAP) assay was used to determine the antioxidant capacity of the extraction solution [14]. The FRAP assay is a simple, commonly used method to evaluate the antioxidant capacity of plant materials and, in particular, fruits [15,16]. Thaipong and colleagues showed the simplicity, speed and high reproducibility of the FRAP method, as well as the highest Pearson correlation value between total antioxidant compounds and total phenolic molecules [17]. The reduction of ferric to ferrous ions at low pH causes a colored ferrous-tripyridyltriazine (TPTZ) complex. The FRAP reagent was freshly prepared as recently reported in Berni et al. [12] by mixing 2040 µL of sodium acetate 300 mM pH 3.6 to 200 µL of TPTZ 10 nM (Sigma Chemical, St. Louis, MO, USA) and 200 µL of ferric chloride 20 mM. At the end, 20 µL of sample were added to the FRAP reagent and then the solution was incubated for 1 h at 37 °C. The solutions were compared to a previously prepared ferric chloride standard curve measured at 593 nm (UV-Vis instrument Shimadzu UV Visible Recording Spectrophotometer UV 160, Shimadzu, Kyoto, Japan). The values were expressed as micromole (µmol) of Fe^2+^ equivalents per gram of fresh weight (µmol Fe^2+^/g FW).

### 2.4. Evaluation of the Phenolic Content

The Folin–Ciocalteu method (F-C method) was performed for the phenolic content determination. The method is described by Berni et al. [12]. Briefly, 0.5 mL of sample was added to 3.0 mL of distilled water and 0.25 mL of F-C reagent (Sigma Chemical, St. Louis, MO, USA). Then, 0.75 mL of saturated sodium carbonate and 0.95 mL distilled water was added to the mixture [18]. The solutions were incubated for 30 min at 37 °C and measured at 765 nm. Results were compared to a previously prepared gallic acid (GA; Sigma chemicals, St. Louis, MO, USA) standard curve. The total phenolic content was expressed as milligrams of gallic acid equivalents per gram of fresh weight (mg GAE/g FW).

### 2.5. Evaluation of the Sugar Content

For quantification and calibration, a standard solution was prepared by dissolving D(+)-fructose, D(+)-glucose, D(+)-sucrose, and poly-D-galacturonic acid methyl ester (to quantify soluble pectin contents via HPLC, as detailed below) in water (Sigma-Aldrich, St. Louis, MO, USA, HPLC grade) for five different concentration levels, viz. 50, 100, 500, 1000, 2500, and 5000 ppm. The sample preparations and HPLC analyses were performed following the methods described by [19]. Five grams of frozen fruits were dissolved in 10 mL of ultra-pure water, homogenized using an Ultra-Turrax and then sonicated for 20 min. The mixture was homogenized once again and centrifuged at 4 °C at 12,000 rpm for 10 min. The supernatant was filtered through a 0.45 µm membrane and then injected into the HPLC loop. For the soluble sugar analysis, a Waters 600 pump E with refraction index detector 2410 was used. The HPLC analysis was performed isocratically with the column Sugar Pak 1 (S5 μm, 250 mm × 4.6 mm i.d.) in ultra-pure water under the following conditions: flow rate = 1.0 μL/min, data rate = 1 pps, run time = 15 min, gain = 1, column heater temperature = 35 °C, sample temperature = 5 °C, pressure = 50 psi, nebulizer: heating (90%) and injection volume = 20.

### 2.6. Statistical Analysis

For each extract, three analytical measurements were performed and the final value was calculated as an average for each sample. Three independent biological replicates were obtained from each variety and the values were reported using the standard deviation (SD). A one-way ANOVA with a Tukey’s post-hoc test was performed on log2 transformed values with IBM SPSS Statistics v19 (IBM SPSS, Chicago, IL, USA). The Pearson correlation coefficient was calculated using a Pearson correlation coefficient calculator (https://www.socscistatistics.com/tests/pearson/Default2.aspx).

## 3. Results

### 3.1. Antioxidant Capacity

The antioxidant capacity values ranged from 97.13 µmol Fe^2+^/g FW to 5.85 µmol Fe^2+^/g FW in the ancient apple varieties. The ‘Solaio’ variety reported the highest antioxidant concentration; also, ‘Campo Pianacce’ and ‘Viale Casetta’ varieties showed interesting values (48.79 and 36.29 µmol Fe^2+^/g FW, respectively, as shown in Table 1). The values obtained for the commercial fruits were 13.51 µmol Fe^2+^/g FW in ‘Red Delicious’ and 11.99 µmol Fe^2^/g FW in ‘Golden Delicious’. Interestingly, 12 out of the 17 ancient varieties studied showed a content of antioxidants that was higher than the commercial ones (Table 1).

### 3.2. Total Phenolic Content

The highest polyphenol contents were found in the varieties ‘Solaio’ and ‘Campo Pianacce’ (8.72 mg GAE/g FW and 2.25 mg GAE/g FW shown in Table 1).

Interestingly, the commercial varieties showed the lowest values: 0.30 mg GAE/g FW for ‘Golden Delicious’ and 0.26 mg GAE/g FW for ‘Red Delicious’ (Table 1). To evaluate the contribution of polyphenols to the total antioxidant capacity, the Pearson correlation coefficient was calculated. It showed a positive correlation (*r* = 0.8803). 

### 3.3. Soluble Sugar and Pectin Contents

As shown in Table 2, the ancient varieties displayed different concentrations, both among themselves and among the commercial ones. As expected, the most prominent soluble sugar in apples was fructose. In the commercial varieties, this sugar was found at the highest concentrations with approximately the same values (‘Golden Delicious’ 69.14 mg/g FW and ‘Red Delicious’ 64.46 mg/g FW). Notably, fructose was in high concentrations also in the ancient varieties ‘Tre Colli’, ‘Ficareto’ and ‘Strada Pianacce’ (63.58, 59.14, and 55.29 mg/g FW, respectively; Table 2), while in ‘Vecchio Pollaio’, and ‘Gialla Pianacce’, the lowest values were obtained (16.31 and 15.52 mg/g FW; Table 2). Differently from fructose, the highest values of sucrose were measured in two ancient varieties: ‘Casolana’ with 61.34 mg/g FW and ‘Ficareto’ with 53.5 mg/g FW (Table 2). 

Concerning glucose, the highest values were measured in ‘Viale Casetta’ (31.69 mg/g FW), ‘Solaio’ (30.72 mg/g FW) and ‘Red Delicious’ (30.11 mg/g FW) and the lowest was observed in ‘Casolana’ (1.96 mg/g FW). Commercial varieties resulted in much higher concentrations, as compared to most ancient fruits, as reported in Table 2. 

The HPLC analysis also showed a high concentration of xylitol in all the varieties studied (Table 2). ‘Rossa Casetta’, ‘Solaio’ and ‘Viale Casetta’ showed the highest xylitol contents (13.79, 13.70, and 13.68 mg/g FW, respectively) and 14 out of 17 ancient varieties displayed higher concentrations, as compared to the commercial counterparts (‘Golden Delicious’ 2.31 mg/g FW and ‘Red Delicious’ 1.29 mg/g FW; Table 2). 

Finally, the chromatographic method used revealed the presence of fibers in overall high concentration. In particular, pectins were detected. Fourteen out of 17 apple varieties showed higher values than commercial ones, particularly ‘Solaio’, ‘Podere Pianacce’ and ‘Ficareto’ varieties (19.72, 17.38, and 17.06 mg/g FW, respectively; Table 2). ‘Rossa Casetta’, ‘Viale Casetta’ and ‘Gialla Pianacce’ reported instead the lowest values (4.05, 4.26, and 4.34 mg/g FW, respectively; Table 2). The commercial apples ‘Red Delicious’ and ‘Golden Delicious’ displayed relatively low values of 5.14 and 6.46 mg/g FW, respectively (Table 2).

## 4. Discussion

Diet plays an important role in human health: the consumption of specific types of food (fruits and vegetables) has been linked to the prevention of chronic diseases [20]. Functional foods, i.e., those foods that have the added value of exerting a positive effect on health, are rich in nutraceuticals (mainly phytochemicals produced by the plant secondary metabolism, such as the phenylpropanoid pathway), which contribute towards mitigating problems related with, e.g., the gastrointestinal tract [21] and prevent chronic diseases, such as type 2 diabetes [22]. The consumption of functional foods improves the antioxidant and anti-inflammatory responses of the organism and helps fight cardiovascular diseases and cancer [21]. 

We here report on the nutraceutical content of 17 ancient apples from the province of Siena in Tuscany and compare the values obtained with those measured in two commercial varieties. Twelve of the 17 ancient fruits displayed antioxidant capacity values that were higher than the commercial apples (Table 1) and, interestingly, a high Pearson coefficient was calculated when correlating the antioxidant capacity with the total polyphenol contents. Polyphenols were found in higher quantities in all the ancient apples here studied (Table 1).

The results presented show that the ancient apples from Siena have a remarkably interesting antioxidant potential, which motivates their use as functional foods. As we recently discussed, the valorization of ancient regional varieties grown by exploiting the local soil (and associated microbiota) can greatly contribute to boosting the regional economy and favors the manufacture of fully traceable products with a minimal C footprint [9]. The recovery of such historical fruit tree species diversifies, at a local level, the consumers’ choice of fruits and also contributes to the restoration of regional habitats.

Our results are even more interesting if one considers the data about the sugars and fibers. While the commercial fruits displayed among the highest glucose levels, some ancient varieties from Siena, such as ‘Strada Pianacce’ and ‘Casolana’, had extremely low values (Table 2). 

Carbohydrates are the main source of energy for the human body. They are, therefore, an essential component of the diet, but a high intake can lead to various problems and diseases, such as hyperglycemia and diabetes. 

The apple fruit can provide sugars for about 12–18% of its weight [23]. Therefore, determining the sugar classes contained is relevant for nutraceutics. The dominant sugar is fructose and its concentrations were higher in the commercial fruits due to the culture conditions used (e.g., specific fertilization regimes) to meet market demands of yield. 

The overall lower presence of glucose in the ancient fruits indicates a potential value of the Tuscan apples in hypoglycemic diets. The higher presence of xylitol in the ancient fruits confirms this, since this sugar has a much lower glycemic index than sucrose and is metabolized independently of insulin [24]. 

A further element in favor of the use of ancient Tuscan apples as functional foods is the content of fibers (pectins): a diet rich in fibers has been associated with a reduced risk of colorectal cancer [25]. Pectins are interesting for their gelling and emollient properties and they are useful in the regulation of intestinal functions, by preventing the reabsorption of bile acids, thus favoring their elimination [26].

The regional cultivation of ancient plants represents an innovative agricultural strategy relying on the exploitation of local natural resources and has a beneficial agronomical impact from an ecological standpoint. In order to cope with the progressive land loss observed in the last years [27] due to the inexorable industrialization, plant varieties thriving in environments with minimal human input (i.e., fertilization, irrigation) are important resources [9,11].

In terms of sustainable agricultural development, wild lands with minimal human intervention preserve the local microbiota which developed in equilibrium with the local microhabitat and soil properties. Ancient plants have established a perfect synergistic relationship with the local microbiota and soil and such a condition is likely contributing to the higher resilience to exogenous stresses observed in these varieties. 

Drought is a major environmental stress compromising agricultural production worldwide and affecting the yield of crops. It is known that non-commercial plant varieties, such as landraces, show enhanced drought tolerance (recently reviewed in [11]), thereby attracting much interest in terms of optimized agricultural programs relying on less water input. 

Last but not least, the high content of antioxidants of ancient plant varieties [9,12] can determine a higher resistance to biotic stresses, thanks to the enhanced production of phenolics or terpenoids, which have a protective effect against pests. This can favor a decrease in the use of pesticides. Therefore, an agricultural management based on the cultivation of locally adapted ancient varieties contributes not only to the preservation of the local biodiversity, but has also a beneficial ecological impact, since less water and pesticides may be used. 

## 5. Conclusions

Our study confirms and strengthens the previously reported data on the nutraceutical value of ancient Tuscan plant varieties. We provide here a case study on apples and show that the ancient fruits recovered across the province of Siena represent a rich source of phenolics with antioxidant activity, fibers and sugars with low glycemic index, notably xylitol. Their use in food products, either fresh or processed, is interesting and such ancient apple fruits are an alternative (and in the case of some varieties here analyzed) superior source of nutraceuticals and fibers. Future studies should aim at characterizing, at a molecular level, the genes/enzymes acting in secondary metabolic pathways in such ancient apple varieties.

## Figures and Tables

**Table 1:** The table shows the values (±SD) of antioxidants (expressed as µmol Fe^2+^/g FW) and polyphenols (expressed as mg GAE/g FW) in the ancient and commercial apples studied. Different letters indicate statistically significant differences among values (*p* < 0.05).

Variety Name	Total Antioxidants	Total Polyphenols
Solaio	97.13 ± 4.94^l^	8.72 ± 2.46^f^
Campo Pianacce	48.79 ± 0.97^k^	2.25 ± 0.06^e^
Viale Casetta	36.29 ± 3.51^j^	1.87 ± 0.04^de^
Gialla Pianacce	28.34 ± 2.25^ij^	1.35 ± 0.07^cde^
Tre Colli	22.87 ± 0.58^hi^	0.88 ± 0.16^cde^
Ancaiano	19.90 ± 1.62^gh^	1.29 ± 0.08^cde^
Piatta Cantine	18.98 ± 1.12^fgh^	1.21 ± 0.03^cde^
Filare Delle Pianacce	18.47 ± 0.55^fgh^	4.25 ± 0.15^e^
Tocchi	18.15 ± 1.45^fgh^	1.86 ± 0.01^de^
Rossa Casetta	16.51 ± 0.85^efg^	0.67 ± 0.07^abc^
Ficareto	15.55 ± 1.56^defg^	1.09 ± 0.10^cde^
Rugginosa Delle Pianacce	14.81 ± 0.16^cdef^	0.80 ± 0.04^bcd^
Red Delicious	13.51 ± 0.35^cde^	0.26 ± 0.01^a^
Vecchio Pollaio	13.43 ± 1.74^cde^	1.10 ± 0.03^cde^
Strada Pianacce	13.29 ± 0.62^bcde^	0.66 ± 0.06^abc^
Golden Delicious	11.99 ± 0.89^bcd^	0.30 ± 0.01^ab^
Podere Pianacce	11.35 ± 1.13^bc^	0.74 ± 0.01^bcd^
Sotto Muro Casetta	9.98 ± 0.12^b^	0.60 ± 0.21^abc^
Casolana	5.85 ± 1.65^a^	0.59 ± 0.20^abc^

**Table 2:** The table shows the values (±SD) of free sugars and pectin (expressed as mg/g FW) in the ancient and commercial apples studied. Different letters indicate significant differences among values (*p* < 0.05).

Variety Name	Glucose	Fructose	Sucrose	Xylitol	Pectins
Solaio	30.72 ± 0.67^l^	48.45 ± 0.26^f^	13.13 ± 0.07^b^	13.70 ± 0.04^k^	19.72 ± 0.06^l^
Campo Pianacce	13.58 ± 0.15^gh^	43.30 ± 0.08^e^	16.96 ± 0.06^c^	3.72 ± 0.05^f^	13.41 ± 0.21^i^
Viale Casetta	31.69 ± 1.57^l^	51.98 ± 0.49^g^	33.36 ± 0.12^i^	13.68 ± 0.04^k^	4.26 ± 0.11^ab^
Gialla Pianacce	17.86 ± 0.28^ij^	15.52 ± 0.32^a^	20.38 ± 0.22^e^	2.61 ± 0.05^d^	4.34 ± 0.07^b^
Tre Colli	17.29 ± 0.34^i^	63.58 ± 0.51^j^	42.35 ± 0.31^l^	6.41 ± 0.32^j^	11.71 ± 0.25^h^
Ancaiano	20.54 ± 0.60^k^	43.59 ± 0.32^e^	20.19 ± 0.08^e^	5.47 ± 0.14^i^	11.86 ± 0.07^h^
Piatta Cantine	19.34 ± 0.34^jk^	49.66 ± 0.20^f^	11.96 ± 0.12^a^	1.01 ± 0.06^a^	9.11 ± 0.12^f^
Filare Delle Pianacce	12.94 ± 0.08^fg^	43.40 ± 0.32^e^	25.03 ± 0.13^f^	4.06 ± 0.09^fg^	13.51 ± 0.05^i^
Tocchi	13.49 ± 0.44^g^	51.68 ± 2.15^g^	41.34 ± 0.20^k^	4.91 ± 0.05^hi^	16.04 ± 0.09^j^
Rossa Casetta	11.93 ± 0.06^f^	51.98 ± 0.62^g^	33.31 ± 0.17^i^	13.79 ± 0.19^k^	4.05 ± 0.16^a^
Ficareto	9.82 ± 0.15^e^	59.14 ± 0.30^i^	53.57 ± 0.31^m^	4.77 ± 0.11^h^	17.06 ± 0.14^k^
Rugginosa Delle Pianacce	14.75 ± 0.16^h^	48.44 ± 0.29^f^	20.21 ± 0.04^e^	4.01 ± 0.07^fg^	12.15 ± 0.16^h^
Red Delicious	30.11 ± 0.42^l^	64.46 ± 0.48^j^	11.81 ± 0.03^a^	1.29 ± 0.09^b^	5.14 ± 0.12^c^
Vecchio Pollaio	8.54 ± 0.08^d^	16.31 ± 0.18^b^	26.55 ± 0.27^h^	3.14 ± 0.07^e^	15.31 ± 0.18^j^
Strada Pianacce	4.50 ± 0.26^b^	55.29 ± 0.29^h^	25.97 ± 0.07^g^	2.10 ± 0.07^c^	10.98 ± 0.16^g^
Golden Delicious	20.44 ± 0.79^k^	69.14 ± 0.36^k^	17.34 ± 0.17^d^	2.31 ± 0.14^c^	6.46 ± 0.26^d^
Podere Pianacce	5.37 ± 0.13^c^	49.68 ± 0.17^f^	32.75 ± 0.10^i^	2.27 ± 0.04^c^	17.38 ± 0.23^k^
Sotto Muro Casetta	9.31 ± 0.26^e^	35.44 ± 0.21^d^	37.63 ± 0.18^j^	4.28 ± 0.31^g^	7.06 ± 0.07^e^
Casolana	1.96 ± 0.09^a^	31.71 ± 0.23^c^	61.34 ± 0.18^p^	3.99 ± 0.03^fg^	9.32 ± 0.18^f^
